# Disparities in Children’s Family Experiences by Mother’s Socioeconomic Status: The Case of Finland

**DOI:** 10.1007/s11113-018-9485-1

**Published:** 2018-08-14

**Authors:** Marika Jalovaara, Gunnar Andersson

**Affiliations:** 1 Department of Social Research, University of Turku, 20014 Turku, Finland; 2 Demography Unit, Stockholm University, 106 91 Stockholm, Sweden

**Keywords:** Socioeconomic status, Education, Lone parenthood, Divorce, Child outcomes, Finland

## Abstract

A well-known argument claims that socioeconomic differentials in children’s family structures have become increasingly important in shaping child outcomes and the resources available to children in developed societies. One assumption is that differentials are comparatively small in Nordic welfare states. Our study examines how children’s experiences of family structures and family dynamics vary by their mother’s educational attainment in Finland. Based on register data on the childbearing and union histories of women in Finland born from 1969 onwards, we provide life-table estimates of children’s (*N* = 64,162) experiences of family dissolution, family formation, and family structure from ages 0–15 years, stratified by mother’s education level at the child’s birth. We find huge socioeconomic disparities in children’s experiences of family structures and transitions. Compared to children of highly educated mothers, children of mothers with low levels of education are almost twice as likely to be born in cohabitation and four times as likely to be born to a lone mother. They are also much more likely to experience further changes in family structure—particularly parental separation. On average, children of low-educated mothers spend just half of their childhood years living with both their parents, whereas those of high-educated mothers spend four-fifths of their childhood with both parents. The sociodemographic inequalities among children in Nordic welfare states clearly deserve more scholarly attention.

**Electronic supplementary material:**

The online version of this article (10.1007/s11113-018-9485-1) contains supplementary material, which is available to authorized users.

## Background

Since the 1960s, affluent democracies have witnessed delays and decreases in marriage and entry into parenthood, combined with increases in non-marital cohabitation, non-marital childbearing, separation, and divorce. These family changes, commonly referred to as the second demographic transition (SDT), have also altered the family structures and dynamics experienced by children (Surkyn and Lesthaeghe 2004; Sobotka and Toulemon 2008; Thomson 2014). Some of the developments, including those related to parental separation and non-marital childbearing, imply losses in the resources available to children (McLanahan 2004; McLanahan and Jacobsen 2015). A large volume of research suggests that children who do not live with both of their parents fare worse on a variety of outcomes, such as educational achievement, socio-emotional development, psychological well-being, health, and socioeconomic achievement in adulthood (Amato 2000, 2010; McLanahan et al. 2013; McLanahan and Jacobsen 2015; Härkönen et al. 2017; Erola and Jalovaara 2017). Many children are born to lone mothers, but in most cases, lone parenthood results from parental separation (Andersson et al. 2017). Many of these children experience their parents’ partnering and repartnering. A growing literature focuses on children’s experience and child outcomes of stepfamily formation, and parental parentering and repartnering (see Thomson and McLanahan 2012; Turunen 2013; Williams et al. 2013; Mariani et al. 2017; Erola and Jalovaara 2017).

Increasing research attention has been directed towards the “diverging destinies” of children born to diverse family contexts (McLanahan 2004; McLanahan and Percheski 2008; McLanahan and Jacobsen 2015). Children born to low-educated mothers are increasingly likely to experience family changes and structures that are associated with a loss of resources, including their parents’ early and non-marital family formation, parents’ union dissolution, lower levels of father involvement, and the parents’ weaker labor market attachment. The opposite holds true for children born to highly educated mothers who tend to benefit from their mother’s later and better-planned family formation, their father’s higher level of involvement, and their parents’ greater union stability and stronger position in the labor market. These unevenly distributed family experiences of children born to disadvantaged and advantaged families have the potential to increase differences in children’s well-being and chances in life (ibid.). The situation for the former thus reflects the accumulation of disadvantages in which an unfavorable relative position produces further relative losses over the life course and across generations, while the latter experience the opposite processes of cumulative advantage, whereby a favorable position becomes a resource that produces further gains (DiPrete and Eirich 2006).

McLanahan’s observations were mostly based on U.S. data, but similar differences and developments have been observed in many European countries as well, although the trends and magnitude of group differences vary (see McLanahan 2004; Kennedy and Thomson 2010; McLanahan and Jacobsen 2015; Härkönen 2017, 2018). For instance, Härkönen (2017) shows that in many countries—including our focal country, Finland—lone motherhood is increasingly associated with low education. Further, several recent studies suggest that affluent societies may have experienced a halt to, and even a reversal of, increases in divorce and declines in fertility (Raley and Bumpass 2003; Kennedy and Ruggles 2014; Anderson and Kohler 2015; Goldscheider et al. 2015; Esping-Andersen and Billari 2015; Jalovaara et al. 2018). These turns are largely ascribed to changes in the behavior of the highly educated and the increasing selectivity of marriage. Taken together, they may lead to further increases in the socioeconomic disparities produced by differential family dynamics.

McLanahan (2004) and McLanahan and Jacobsen (2015), and others have argued that welfare states could and should help reduce inequalities in children’s opportunities. McLanahan (ibid.) claimed that income-tested benefits to single parents, widely in use in the U.S., discourage marriage and employment among low-educated women and thereby contribute to wider socioeconomic disparities. Expectations are that socioeconomic differentials in an array of outcomes would be more narrow in the Nordic welfare regime where state policies are targeted at promoting social and gender equality, and where the state provides welfare support based on a universalistic approach (Esping-Andersen 1999; Kautto et al. 2001; Kvist et al. 2012). In Finland, government policies aimed at decreasing social and gender inequalities include, for instance, extensive parental-leave schemes, strongly subsidized children’s day care, child allowances, tuition-free education and student allowances, low-cost or free public health care, individualized taxation, unemployment benefits, and housing allowances (see Niemelä and Salminen 2006). Comparatively low-income differentials and low poverty rates are well-known features of the Nordic countries (Kautto et al. 2001; Kvist et al. 2012). Based on the Gini index estimate, which was 26.8 in 2014, Finland is among the most equal nations in the world (World Bank 2018). Finland has also long ranked very high in PISA assessments of excellence and equity in education, although its relative position has recently deteriorated (Reinikainen 2012; OECD 2016). However, whether welfare policies can in fact influence children’s access to a stable two-parent family is unclear. Previous research reports notable sociodemographic disparities in the Nordic countries; these disparities may be smaller than those in the U.S., but increases over time have been observed in Nordic countries as well (Kennedy and Thomson 2010; Härkönen 2017). In line with Cohen (2015) and Härkönen (2017, 2018), we propose that the role of policies is mainly to keep differences in family experiences from translating into disparities in children’s well-being and chances in life.

Almost all previous research on socioeconomic differentials in family dynamics in Finland and the other Nordic countries concerns adults. These findings show that compared to low-educated women, highly educated women are more likely to marry, more likely to have their children in marriage, much less likely to have a child outside of a coresidential partnership, and less likely to separate or divorce (Hoem 1997; Holland 2013; Jalovaara and Fasang 2015; Schnor and Jalovaara 2017; Jalovaara and Kulu 2018). While gradients among adults provide suggestive evidence for children’s experience, family transitions look somewhat different when one adopts a child perspective, mainly because the latter also reflects differences in the timing and quantum of childbearing between adults in different family situations (Thomson et al. 2014).

In the case of Nordic countries, non-marital cohabitation is a factor of particular significance (see also Kennedy and Thomson 2010). American discussion, including the argument made by McLanahan (2004) and McLanahan and Jacobsen (2015), tend to stress the role of marriage in securing children’s access to a stable family life. In the Nordic countries, cohabitation is a more established union type, and therefore, one could expect smaller differences in outcomes such as union stability between cohabitation and marriage. However, we may still anticipate differences in children’s experiences because previous research reports that separation rates are higher for cohabiting than for married parents in the Nordic countries as well (Andersson 2002; Heuveline et al. 2003; Andersson et al. 2017).

Maternal socioeconomic status is often measured by mother’s level of educational attainment, an approach we adopt here as well. We opt for this approach because education is a good proxy of a person’s human capital and a predictor of his or her labor market position, earnings, and wealth. It reflects opportunities and resources available to adults, which also affect their children and are transferred to them in processes of socioeconomic inheritance (Korupp et al. 2002; Erola et al. 2016). The different family demographic behaviors of women at different educational levels may also reflect their attitudes and values, as well as their broader life-course opportunities and constraints. Further, factors related to maturity matter, as educational attainment is strongly related to the ages women have their children. In the SDT concept, a central argument is that highly educated women and men are, owing to their less traditional attitudes, forerunners in beginning new family behaviors (Surkyn and Lesthaeghe 2004). However, more recent research finds that liberal attitudes (see e.g., Gubernskaya 2010) are often not translated into more behaviors such as separating or having children outside of marriage (Sobotka 2008).

Finland and other European countries (Vincent-Lancrin 2008) have also witnessed a remarkable educational expansion. The developments have been particularly strong for women. In our study cohorts (mothers were born in 1969 onwards), the majority of women have eventually completed high (tertiary) education, and less than one-tenth of women have completed no degrees beyond the compulsory basic level. At the same time, the labor market position of low-educated women and men has weakened. This phenomenon was particularly pronounced in the wake of the recession in the 1990s that was exceptionally severe in Finland and gave additional input to the restructuring of the economy from industrial to other higher-skilled sectors of employment (Asplund and Maliranta 2006; Hannikainen and Heikkinen 2006). Educational differences in current unemployment levels are noticeable. In 2016, the unemployment rate was 16% for women and men with less than a secondary education (i.e., low-educated), 10% for those with a completed secondary education (i.e., medium educated), and 6% for those with a degree at tertiary level (i.e., highly educated) (Statistics Finland 2018a). A recent Nordic comparison (Jalovaara et al. 2018) reported increasing social inequalities in family demographic outcomes as well, as measured by cohort fertility and ultimate childlessness. The authors argued that the less educated have become increasingly marginalized also with regard to family formation, with persistent educational disparities among men and new disparities evolving among women. A recent study (Härtull et al. 2017) reports on widening socioeconomic disparities in terms of economic resources available for children in Finland.

In the current study, we proceed to examine how children’s experience of family dynamics and family structure varies by their mother’s educational attainment in the context of Finland. We base our study on population register data on women born in 1969 and later who were observed during a six-year long study window ending in 2009, which is the last year for which we have data. Based on the childbearing and union histories of these women, we provide life-table estimates of their children’s experience of different family structures and family transitions at ages zero to fifteen during 2003–2009. We thus apply a synthetic-cohort approach to describe the family circumstances and transitions children experience during a given calendar period. The purpose of focusing on the most recent period for which we have data is to give an account of the current and most recent state of affairs in family dynamics in Finland. All analyses are stratified by the mother’s educational attainment at the time of the birth of each child.

## Data and Methods

The current study builds on previous projects on life-table representations of family dynamics in Europe based on data from the fertility and family surveys (FFS) (Andersson 2002; Andersson and Philipov 2002) and the generations and gender surveys (GGS) (Andersson et al. 2017) from a range of countries in Europe and beyond. We use data from Finland that are arranged in the same way and exposed to exactly the same research design and methods of analysis as those applied in these previous studies. In all of these studies, longitudinal histories of women’s childbearing, union formation, and union dissolution events are used as the basis to construct life-table estimates on the cumulative proportions of women, men, and children who experience different family events. The purpose of our design is to achieve maximum comparability with these earlier studies and their life-table estimates for other countries in Europe and North America. There are some differences in the current study from previous work. First, we have a much larger sample than many studies relying on surveys as our data are derived from the register data for Finland. Second, our analyses focus on the experiences of children rather than adults or couples. Finally, our results are stratified by maternal education to illustrate the importance of socioeconomic differences in children’s family transitions. Finland has not participated in the GGS program, but we will demonstrate that much of the same data can be drawn from population and administrative registers. This similarity in data holds true for Finland in particular, where data on registered domiciles are available for a longitudinal depth of up to three decades, which allow for the inference of histories of non-marital cohabitation as well as those of changes in civil status and childbearing events. When using register data, the immediate costs of data compilation can be kept very low (amounting to a few days of additional data coding if data are already in use). Another positive feature is the opportunity to link data to reliable data on other related factors, such as histories of educational attainment. Finally, some typical problems inherent in sample surveys are avoided. One of these problems is selective non-response and attrition (e.g., those who have recently divorced are often less likely to participate in family surveys; Andersson et al. 2017). Another problem avoided in the current study setup is the impact of subjective interpretations of different family situations. For instance, partners who spend much time together but continue to have their own apartments may be inclined to report themselves as living together in a survey, while others do not. These biases in surveys tend to produce underestimates of union instability and non-union childbearing. The situation in which panel attrition is much higher among low-educated survey respondents (Kennedy and Thomson 2010) tends to aggravate these problems for certain sub-strata of the population. Weaknesses of register data are largely related to the fact that certain aspects of family relations, such as children’s contact with non-resident parents, remain unobserved.

### Data

We use data that were compiled at Statistics Finland by linkages of data from different register sources. The extract used in the current study is taken from a random 11% sample^1^ of persons born between 1940 and 1995 who had been recorded as residents in the Finnish population between 1970 and 2010, although not necessarily during the entire period. Since 1987, women and men’s union histories have covered not only marriage but also cohabitation. A cohabiting couple is defined as a man and a woman who are registered as domiciled in the same dwelling for over 90 days, who are not close relatives (siblings or a parent and a child, for example) or married to each other, and whose age difference is no more than 20 years. The rule on age difference does not apply if the couple has shared children.^2^ Limitations are that non-cohabiting “Living Apart Together” relationships remain unnoticed and same-sex cohabitations cannot be determined, but these types of relationships are relatively few in our analyses, which are limited to relationships formed by those with children.

We focus on children to women born between 1969 and 1993. The 1969 cohort of mothers is the oldest to have full histories of all coresidential unions beginning from the year of their 18th birthday and stretching to age 40. Analyses are further confined to women who were members of the Finnish population when they turned 18. Of these women, 97% were born in Finland. Women who had died or emigrated from Finland before the last date for which we have data were omitted from the analyses, which would have been the situation had a comparable sample survey been conducted in 2009 (Andersson and Sobolev 2013). Childbearing and union histories until September 2009 were available to us. The childbearing histories provide the study population of children that are used for our analyses. The children who contribute to our life-table estimates were born between April 1985 and September 2009.

Our data on mothers’ educational attainment are based on Statistics Finland’s register of completed degrees. In the present analyses, we use the highest education attained by the mother by the time of her child’s birth (monthly precision). This approach was chosen to avoid problems inherent in any anticipatory analysis (Hoem and Kreyenfeld 2006): education measured at an older child age could be affected by different family and educational dynamics after childbirth. We distinguish between low (basic: ISCED97 1–2), medium (secondary: ISCED97 3–4), and high (tertiary: ISCED97 5–6) educational level. The low educated comprise persons for whom no data on post-comprehensive, non-compulsory education are registered.

The analyses cover 64,162 children. Of them, 18% (*N* = 11,251) had a low-educated mother, 44% (*N* = 28,398) a medium-educated mother, and 38% (*N* = 24,513) a high-educated mother. Thus, the mothers’ educational level is quite high on average, although some women continued their education and completed even higher degrees at a later stage of the life course. The educational expansion does not influence our analysis, as our oldest cohorts were already highly educated and subsequent increases have been relatively small.

### Methods

The life-table estimates are based on age- or duration-specific transitions of different kinds that are observed during a person’s childhood. The transitions refer to different family-formation and dissolution events as registered for the child’s mother. The resulting life tables are constructed on the basis of a synthetic cohort that covers the state of affairs in family dynamics in Finland during the 6-year period from October 2003 to September 2009, which is the last month for which we have data. As is always the case with synthetic-cohort measures, the results describe the demographic patterns that would have arisen if the age- or duration-specific transition probabilities prevailed while a cohort of children passes through those ages or durations (see Andersson and Philipov 2002; Andersson et al. 2017).

Children’s life courses are followed from birth until the life-course event of interest occurs, or if it does not occur, until age fifteen or censoring in September 2009. Parental unions cease to be observed at the death of the mother’s partner. Observations are also censored if the child dies and, in most analyses, if a child is observed as living separately from the mother prior to age fifteen. All duration spells are based on data with a precision of 1 month. Spells and transitions that refer to periods prior to October 2003 do not contribute directly to the life-table estimates we produce.

We do not incorporate additional information on fathers’ union histories and other characteristics. The great majority of children who do not live with both of their parents, live with their mother. Thus, mothers’ union histories better represent their children’s family experiences.

In what follows, we provide an overview of Finnish children’s (i) family status at birth for children born during 2003–2009, (ii) experience of family dissolution events by means of life-table estimates of the cumulative fractions ever living outside a two-parent family, (iii) experience of different family-formation events of children born to a lone mother, two cohabiting parents, and those whose parents separate during childhood, and (iv) childhood time in different family constellations. We also provide the complete single-year life-table tabulations in an Electronic Supplementary Material.

## Results

We start by examining the family structures to which children are born and continue with children’s experiences of parental union dissolution and of ever living outside of a family that consists of both their parents. We then proceed with the experience of any union-formation events among non-partnered mothers. The analysis concludes with a summary statistic on the proportion of time that the children spent in various family types during childhood. All analyses are stratified by maternal education at the birth of the focal child.

### Family Type at Birth

Table 1 shows the relative distribution of births that occurred while the mother was living in cohabitation, marriage, or neither. Of all children, almost nine out of ten were born to a mother who was cohabiting or married at the time of the birth. One-third of all children were born in cohabitation, and the majority were born in marriage.

**Table 1:** Family type at birth by maternal education among children born in Finland, 2003–2009, percentage distributions. *Source* Finnish register data, authors’ own calculations

Family type, %	Maternal education			
	Low	Medium	High	All
Born to lone mother, in total (A)	27	12	7	12
Born to mother never in union	12	4	2	4
Born after union disruption	14	9	5	8
Born in marriage (B)	31	48	69	55
Born in cohabitation (C)	43	39	24	33
Total (A + B + C)	100	100	100	100
*N*	11,251	28,398	24,513	64,162

The differences by maternal education are tremendous. Compared to children of highly educated mothers, children of low-educated mothers were almost twice (1.8 times) as likely to be born to a cohabiting mother and four times as likely to be born to a lone mother. In turn, the large majority (about 70%) of children of highly educated mothers were born in marriage, whereas less than one-third of children born to low-educated mothers were born in marriage.

### Experience of Family Dissolution

Table 2 focuses on children who were born in a coresidential union and shows (as cumulative percentages) the extent to which a child has, by certain ages, left or lost his or her original family of two parents. The event of family dissolution includes parental separation, the death of the father, and the child’s own move away from his or her parents. The vast majority of such events occurs due to parental separation; the other two events are very rare at these young ages. Since the likelihood of union dissolution is known to vary by civil status, we show the results separately for children born in any union, in cohabitation, and in marriage.

**Table 2:** Cumulative percent of children in Finland ever out of parental union, by age of child and maternal education, 2003–2009; children born in any union, in cohabitation, and in marriage. *Source* Finnish register data, authors’ own calculations

Age	Maternal education			
	Low	Medium	High	All
Children born in any union (cohabitation or marriage)				
1	10	3	1	3
3	27	11	5	10
6	43	21	12	20
9	53	30	18	28
12	61	36	24	35
15	65	42	29	41
* N*	8289	24,996	22,842	56,127
Children born in cohabitation				
1	14	5	2	5
3	34	15	9	16
6	51	28	17	29
9	61	38	25	39
12	67	46	31	46
15	71	52	35	52
*N*	4782	11,271	6027	22,080
Children born in marriage				
1	5	2	1	1
3	17	7	4	6
6	33	15	10	14
9	42	23	16	21
12	51	28	21	27
15	56	33	27	33
*N*	3507	13,725	16,815	34,047

Table 2 shows that of all children born in a union, 41% had seen their original family dissolved by age fifteen. In addition, marriage does matter: among children born in marriage this proportion was one-third, while for those born to cohabiting parents, it was more than half. However, the disparities by maternal education are much more remarkable; this holds true especially at young child ages. For children of low-educated mothers, the likelihood of experiencing parental family dissolution is remarkably high: 43% of children of low-educated mothers had experienced family dissolution already by 6 years of age, and by age fifteen, the portion was as high as two-thirds. This finding can be compared to the same statistics of only 12 and 29%, respectively, among children of highly educated mothers. The relative differences by educational status are similar regardless of whether the child was born in cohabitation or marriage. However, given the finding that with low maternal education, a larger proportion of children are born in cohabitation, the greater fragility of cohabitations compared to marriages helps contribute to the overall disparity in children’s family outcomes.

### Summary of Experience of Living Outside Parental Union

Table 3 brings together the processes covered in Tables 1 and 2. It shows the cumulative percentage of children who had ever lived outside the union of their parents at various child ages. Children who are born to a lone mother enter this stage at birth (age 0), and children born to a partnered mother who experiences parental union dissolution gradually add to this initial proportion.

**Table 3:** Cumulative percent of children in Finland ever out of union of their parents, by age of child and maternal education, 2003–2009. *Source* Finnish register data, authors’ own calculations

Age	Maternal education			
	Low	Medium	High	All
1	34	15	8	14
3	46	22	12	21
6	59	31	18	29
9	66	39	24	37
12	71	44	29	43
15	74	49	34	48
*N*	11,251	28,398	24,513	64,162

Almost half of all children in Finland had, at some point during childhood, lived outside any parental union. As seen from the results presented thus far, the gaps in children’s family experiences by maternal education are tremendous, and they are the widest at younger child ages. By age three, almost half of children born to low-educated mothers had lived outside a parental union, while only 12% of children with a high-educated mother had lived outside a parental union. At ages 0–3 years, the comparable fractions were more than four times higher for children born to a low-educated mother than for those born to a high-educated one. By age fifteen, the overall proportion reached three-quarters of children of low-educated mothers, compared to one-third of those with a highly educated mother.

### Experience of Mother’s Union Formation and Re-formation

Table 4 focuses on children born to a lone mother and shows the extent to which their mother had formed a coresidential union by given child ages. Of all children born to a lone mother, almost 70% had experienced living in a two-parent family by age fifteen. Children of low-educated mothers were most likely to be born in a lone-parent family and were also the most likely to experience subsequent changes in their family structure. Children of low-educated mothers were almost twice as likely to experience their mother’s union formation than children of high-educated lone mothers. Note that of children born to lone mothers, as many as 16% were found living with a partnered mother within 1 year. While some form a new stepfamily quickly, it is also common that such immediate entry into a coresidential union involves the father moving in with his child.

**Table 4:** Cumulative percent of children in Finland ever in a two-parent family, by age of child and maternal education, 2003–2009; children born to a lone mother. *Source* Finnish register data, authors’ own calculations

Age	Maternal education			
	Low	Medium	High	All
1	20	15	13	16
3	39	31	26	32
6	58	47	34	48
9	70	58	41	58
12	78	64	42	65
15	82	65	42	69
*N*	2962	3402	1671	8035

Table 5 instead focuses on children born in cohabitation and shows the percentages of children who have experienced their parents getting married by given child ages. The estimates are based on a double-decrement competing risk model, with parental separation as the competing event (Andersson and Philipov 2002). The results suggest that the significance of maternal education in relation to marriage formation is, to a large extent, tied to the time shortly after the birth of the child. Of children born to highly educated cohabiting mothers, one-fifth have experienced their parents marrying within 1 year. Among children born to low- and medium-educated cohabiting mothers, this proportion is 10 and 13%, respectively. By their 15th birthday, the majority (64%) of children of highly educated mothers and a minority of those of low-educated cohabiting mothers (41%) had experienced their parents’ getting married.

**Table 5:** Cumulative percent with a married mother, by age of child and maternal education at childbirth, 2003–2009, for children born in cohabitation in Finland; competing-risks life-table method with family dissolution as competing event. *Source* Finnish register data, authors’ own calculations

Age	Maternal education			
	Low	Medium	High	All
1	10	13	19	15
3	24	31	38	32
6	33	45	51	45
9	37	51	59	51
12	40	55	62	54
15	41	57	64	56
*N*	4782	11,271	6027	22,080

Finally, Table 6 displays data on the children who had experienced the dissolution of their parents’ union and shows to what extent they had come to live with their mother and any new maternal partner by various durations of time since the parental union dissolution. It seems that stepfamilies are formed relatively quickly so that within 3 years from the dissolution, four out of ten children had landed in a new stepfamily. The cumulative proportions are highest for children of low-educated mothers, but in this regard, the differences by maternal education are modest.

**Table 6:** Cumulative percent of children ever again in a union, by maternal education and time elapsed since union disruption, 2003–2009, for children in Finland experiencing parental separation. *Source* Finnish register data, authors’ own calculations

Duration (years)	Maternal education			
	Low	Medium	High	All
1	28	25	20	25
3	45	42	36	41
6	62	59	52	58
8	69	66	57	65
10	74	72	63	71
*N*	5636	6715	2698	15,049

### Time Spent in Different Family Types

We conclude our presentation with a crude summary of proportions of time that children in Finland spend in various family types (Table 7). The percentages have been calculated from the states that were observed for the children by different ages (from 0 to 15 years) over the six-year period of this study.^3^ We distinguish between time spent with a lone mother at childbirth and a lone mother after union dissolution. We also show the proportions of childhood time with the two original parents who were married or cohabiting as well as the fractions of time in a new stepfamily that includes the mother. On average, children in Finland spend 69% of their childhood years living with both of their parents. However, the disparities by maternal education are, again, striking. Children of low-educated mothers spend on average almost three times as much of their childhood in a family with a lone mother or living without a mother than do children of highly educated mothers (36% vs. 13% of the total time, respectively). The latter, in turn, spend much more time with both their parents, whether cohabiting or married (81% vs. 51% of the total time at ages 0–15 years, respectively). Compared to children of low-educated mothers, children of highly educated mothers also spend twice as much time living with both parents being married. With regard to time spent in stepfamilies, the differences by socioeconomic status are smaller.

**Table 7:** Percent of time spent in different family types at ages 0–14 years in Finland, 2003–2009, by maternal education. *Source* Finnish register data, authors’ own calculations

	Maternal education			
	Low	Medium	High	All
With lone mother	27	16	11	17
With lone mother, from birth	9	5	3	5
With lone mother, after union dissolution	19	12	8	12
With both parents	51	71	81	69
With both parents in cohabitation	18	16	10	14
With both parents in marriage	33	54	70	55
In step union, with mother	13	9	6	9
With others	9	4	2	5
*N*	11,251	28,398	24,513	64,162

## Conclusions

McLanahan (2004) and McLanahan and Jacobsen (2015) argued that children of low-educated mothers are disproportionately affected by increases in non-marital childbearing and parental separation, which lead to further losses of resources available to these children. The opposite processes are at work among children of highly educated mothers, who tend to benefit from their mother’s later and better-planned family formation, their father’s higher level of involvement, and their parents’ greater union stability and strong labor market position. However, there is some expectation that such a link between maternal education and childhood family structures could be relatively weak in the Nordic countries with greater social and gender equality and more support for parents and children regardless of family type than in other countries.

Our study focused on disparities in children’s experiences of family structures and transitions by maternal educational attainment in Finland. The empirical evidence suggests that there are huge socioeconomic disparities in Finnish children’s experiences of family transitions and family life. The probabilities for children of low-educated women to be born to a lone mother are almost four times as high as for children of high-educated mothers. In addition, if born to a partnered mother, children of low-educated mothers are substantially more likely to experience further changes in family structure—parental separation in particular. As a result, children of low-educated mothers spend a much larger share of their childhood years in lone-parent families than do children of more highly educated mothers. Stepfamily formation can be seen as either contributing to family complexity or as a partial (re)gaining of parental resources. Consistent with previous research from Sweden (Turunen 2011), differences by parental education in entering stepfamilies are less stark.

The socioeconomic disparities in children’s family structures and transitions are largest at the youngest child ages. Consequently, children of low-educated mothers are not only more likely to experience life in a lone-parent family but also tend to land in such families at a much younger age. A notable portion of children born to low-educated mothers start their lives with a lone mother. For the majority of these children, the implication is that they spend little or no time living with their biological father.

Our study also provides evidence of an overall high degree of family instability in Finland. Compared to the percentages in the most recent life-table analyses based on GGS data (Andersson et al. 2017), the proportion of children born to lone mothers in Finland is higher than the proportion in most European countries, similar to that of Russia, but somewhat lower than in the U.S. Births in cohabitation are less common and marital births are more common in Finland than in Sweden and Norway. However, these proportions are similar to those of many other European countries. In Finland, a large number of children experience parental union dissolution, almost reaching the levels observed in the U.S. As mentioned in the data section, the lower levels of family instability and non-union childbearing observed elsewhere in Europe may partially stem from weaknesses attributed to available sample survey data.

Our results show that in Finland, stepfamily formation is a common experience after parental separation as well as among children born to a lone mother. Of children in the latter group, 48% had lived in a two-parent family by age six. However, these levels are even higher in many other European countries and in particular in the U.S., where 69% of 6-year olds born to a lone mother had lived in a two-parent family (Andersson et al. 2017).

Further, we note that parents’ civil status makes a difference in terms of levels of child-family dissolution: one-third of children born in marriage and half of children born in cohabitation witness their parents separate before they turn fifteen. However, the gap is not as large as in the U.S., where the corresponding portion is the same, about one-third, for children born in marriage, but almost three-quarters for those born in cohabitation (Andersson et al. 2017). Previous Finnish research demonstrates very large differentials in union stability between cohabitations and marriages in general (Jalovaara and Kulu 2018). However, from the perspective of children born in those unions, the differences in outcomes are much smaller.

Should researchers and policymakers in Finland be concerned about the diversity in children’s outcomes in terms of family structure and experience? A certain degree of lone parenthood in a society reflects the possibility for women, men, and children to escape detrimental family conditions (McLanahan 2004). However, for children, the experience of living in a family situation other than an intact two-parent family is often associated with different negative outcomes during the life course (Amato 2010; Härkönen et al. 2017), and the finding that lone parenthood is excessively experienced in already-disadvantaged social strata is, in our view, a cause for concern. More generally, the situation reflects that life events that may lead to the loss of resources are more common among those with the fewest resources to begin with and the least means to address any harmful consequences of disruptive family events. This exemplifies the accumulation of disadvantage, in which an unfavorable relative position produces further losses, and contributes to the widening of social and economic inequalities over life courses and across generations (DiPrete and Eirich 2006).

In the Finnish context, women are highly educated on average, and the rates of female labor force participation are among the highest in the world. For instance, in 2016, 80% of mothers of underage children and 82% of all women aged 20–59 were in the labor force (Statistics Finland 2018b). However, lone mothers’ employment rates, which have been high in international comparison, have deteriorated (Härkönen et al. 2016). During the recession in Finland in the 1990s, the employment rates of lone mothers dropped below those of partnered mothers and have not recovered, reflecting the weakened situation of low-educated lone mothers in the labor market. These mothers appear double disadvantaged in that their employment opportunities are restricted not only by their low education but also by their difficulties in combining childrearing and employment (ibid.; Härkönen 2017). Improving this situation may not be easy because low-educated lone mothers have often taken short, youthful, and less-planned routes to parenthood, and their motherhood likely interferes with further educational progress. In the United States, a key reason for socioeconomic disparities in family experience is differences in unintended pregnancy among young women. In Finland, this likely plays a smaller role. For instance, the rates of adolescent childbearing are much lower: in 2014, they amounted to 6.7 births per 1000 women aged 15–19 in Finland, compared to 24.1 per 1000 in the U.S. (World Bank 2018).

Repartnering, which is common among lone mothers, tends to add resources available to children. Previous research suggests that children benefit from stepfathers’ socioeconomic resources (see Erola and Jalovaara 2017). However, educational homogamy also plays a role in partner selection (Mäenpää 2015) and therefore, repartnering may not help ameliorate the socioeconomic disparities that we observe.

McLanahan (2004) presumed that the link between social strata and children’s family structures would be relatively weak in Nordic welfare states. Finland does not fulfill this expectation although, typical of the Nordic countries, many policies are targeted at promoting social and gender equality, and social policies are guided by the principle of universalism. It may be the case that sociodemographic differentials in behavior are larger in Finland than in the other Nordic countries (although Härkönen 2017 reports large educational differences in lone-motherhood prevalence not only in Finland but also in Sweden and Norway). In any case, our study provides little evidence of Nordic social equalities in terms of family demographic outcomes such as parental separation. We do not expect welfare states to affect family demographic behavior directly. Rather, the realistic aim for policymakers is to reduce any detrimental side effects of differential family dynamics on behalf of adults and children and their chances in life (see also Härkönen 2017, 2018; Cohen 2015). To conclude, in recent cohorts, remarkable proportions of children grow up in families headed by a lone mother whose labor market position may be relatively weak. This finding highlights how critical it is to maintain the institutions and practices that help compensate for related losses of resources. Overall, the sociodemographic inequalities among children in Nordic welfare states clearly deserve more policy as well as scholarly attention.

## Electronic supplementary material

Below is the link to the electronic supplementary material.

Supplementary material 1 (DOCX 36 kb)

## Footnotes

1 The data are detailed and rich in information. To protect individual anonymity, Statistics Finland has opted to deliver a sample only.

2 For details on cohabitation inference, see Jalovaara and Kulu (2018).

3 The statistics are derived directly from the distribution of children over family states for the calendar period we study. They are not based on any type of increment–decrement life-table analysis but are standardized for the age composition of children during 2003–2009.
