# Supplementary material for: Disparities in Children’s Family Experiences by Mother’s Socioeconomic Status: The Case of Finland
Source: Popul Res Policy Rev. 2018 Aug 14;37(5):751–68. doi: 10.1007/s11113-018-9485-1 (PMC6267228; doi:10.1007/s11113-018-9485-1)
Supplement: Supplementary file 1 — Supplementary material 1 (DOCX 36 kb) [file 11113_2018_9485_MOESM1_ESM.docx]

Appendix Table 1. Family type at birth by maternal education, relative distribution (%)

|  |  |  | Maternal education | |  |  |
| --- | --- | --- | --- | --- | --- | --- |
| Family type, % | |  | Low | Medium | High | All |
| Born to lone mother, in total (A) | | | 27 | 12 | 7 | 12 |
| - Born to mother never in union | | | 12 | 4 | 2 | 4 |
| - Born after union disruption | | | 14 | 9 | 5 | 8 |
| Born in marriage (B) | |  | 31 | 48 | 69 | 55 |
| Born in cohabitation (C) | |  | 43 | 39 | 24 | 33 |
| Total (A+B+C) | | | 100 | 100 | 100 | 100 |
| N |  |  | 11,251 | 28,398 | 24,513 | 64,162 |

Appendix Table 2. Cumulative percent of children in Finland ever out of parental union, by age of child and maternal education, 2003–2009; children born in any union, in cohabitation, and in marriage

| Children born in any union (cohabitation or marriage) | | | | |
| --- | --- | --- | --- | --- |
|  | Maternal education | |  |  |
| Age | Low | Medium | High | All |
| 0 | 0 | 0 | 0 | 0 |
| 1 | 10 | 3 | 1 | 3 |
| 2 | 20 | 7 | 3 | 7 |
| 3 | 27 | 11 | 5 | 10 |
| 4 | 33 | 15 | 7 | 13 |
| 5 | 39 | 18 | 9 | 17 |
| 6 | 43 | 21 | 12 | 20 |
| 7 | 47 | 24 | 14 | 23 |
| 8 | 50 | 27 | 16 | 25 |
| 9 | 53 | 30 | 18 | 28 |
| 10 | 55 | 32 | 20 | 30 |
| 11 | 58 | 34 | 22 | 33 |
| 12 | 61 | 36 | 24 | 35 |
| 13 | 63 | 38 | 26 | 37 |
| 14 | 64 | 40 | 27 | 39 |
| 15 | 65 | 42 | 29 | 41 |
| N | 8,289 | 24,996 | 22,842 | 56,127 |

(Continued)

| Children born in cohabitation | | | | |
| --- | --- | --- | --- | --- |
|  | Maternal education | |  |  |
| Age | Low | Medium | High | All |
| 0 | 0 | 0 | 0 | 0 |
| 1 | 14 | 5 | 2 | 5 |
| 2 | 27 | 11 | 5 | 12 |
| 3 | 34 | 15 | 9 | 16 |
| 4 | 41 | 20 | 11 | 21 |
| 5 | 47 | 24 | 14 | 25 |
| 6 | 51 | 28 | 17 | 29 |
| 7 | 55 | 32 | 20 | 33 |
| 8 | 58 | 35 | 22 | 36 |
| 9 | 61 | 38 | 25 | 39 |
| 10 | 63 | 41 | 28 | 41 |
| 11 | 65 | 43 | 30 | 44 |
| 12 | 67 | 46 | 31 | 46 |
| 13 | 69 | 48 | 32 | 49 |
| 14 | 71 | 50 | 34 | 51 |
| 15 | 71 | 52 | 35 | 52 |
| N | 4,782 | 11,271 | 6,027 | 22,080 |

| Children born in marriage | | | | |
| --- | --- | --- | --- | --- |
|  | Maternal education | |  |  |
| Age | Low | Medium | High | All |
| 0 | 0 | 0 | 0 | 0 |
| 1 | 5 | 2 | 1 | 1 |
| 2 | 12 | 4 | 2 | 4 |
| 3 | 17 | 7 | 4 | 6 |
| 4 | 23 | 10 | 6 | 9 |
| 5 | 53 | 13 | 8 | 11 |
| 6 | 33 | 15 | 10 | 14 |
| 7 | 62 | 18 | 12 | 16 |
| 8 | 66 | 20 | 14 | 18 |
| 9 | 42 | 23 | 16 | 21 |
| 10 | 74 | 25 | 18 | 23 |
| 11 | 76 | 26 | 20 | 25 |
| 12 | 51 | 28 | 21 | 27 |
| 13 | 81 | 30 | 23 | 29 |
| 14 | 81 | 32 | 24 | 31 |
| 15 | 56 | 33 | 27 | 33 |
| N | 3,507 | 13,725 | 16,815 | 34,047 |

Appendix Table 3. Cumulative percent of children in Finland ever out of union of their parents, by age of child and maternal education, 2003–2009

|  | Maternal education | |  |  |
| --- | --- | --- | --- | --- |
| Age | Low | Medium | High | All |
| 0 | 27 | 12 | 7 | 12 |
| 1 | 34 | 15 | 8 | 14 |
| 2 | 42 | 19 | 10 | 18 |
| 3 | 46 | 22 | 12 | 21 |
| 4 | 51 | 25 | 14 | 24 |
| 5 | 55 | 28 | 16 | 27 |
| 6 | 59 | 31 | 18 | 29 |
| 7 | 61 | 34 | 20 | 32 |
| 8 | 63 | 36 | 22 | 34 |
| 9 | 66 | 39 | 24 | 37 |
| 10 | 67 | 41 | 26 | 39 |
| 11 | 69 | 42 | 28 | 11 |
| 12 | 71 | 44 | 29 | 43 |
| 13 | 73 | 46 | 31 | 45 |
| 14 | 74 | 48 | 32 | 46 |
| 15 | 74 | 49 | 34 | 48 |
| N | 11,251 | 28,398 | 24,513 | 64,162 |

|  | Maternal education | |  |  |
| --- | --- | --- | --- | --- |
| Age | Low | Medium | High | All |
| 0 | 0 | 0 | 0 | 0 |
| 1 | 20 | 15 | 13 | 16 |
| 2 | 30 | 26 | 22 | 26 |
| 3 | 39 | 31 | 26 | 32 |
| 4 | 46 | 38 | 30 | 39 |
| 5 | 53 | 43 | 32 | 43 |
| 6 | 58 | 47 | 34 | 48 |
| 7 | 62 | 51 | 35 | 51 |
| 8 | 66 | 54 | 39 | 55 |
| 9 | 70 | 58 | 41 | 58 |
| 10 | 74 | 61 | 42 | 62 |
| 11 | 76 | 62 | 42 | 64 |
| 12 | 78 | 64 | 42 | 65 |
| 13 | 81 | 64 | 42 | 67 |
| 14 | 81 | 65 | 42 | 68 |
| 15 | 82 | 65 | 42 | 69 |
| N | 2,962 | 3,402 | 1,671 | 8,035 |

Appendix Table 4. Cumulative percent of children in Finland ever in a two-parent family, by age of child and maternal education, 2003–2009; children born to a lone mother

Appendix Table 5. Cumulative percent with a married mother, by age of child and maternal education at childbirth, 2003–2009, for children born in cohabitation in Finland; competing-risks life-table method with family dissolution as competing event

|  | Maternal education | |  |  |
| --- | --- | --- | --- | --- |
| Age | Low | Medium | High | All |
| 0 | 0 | 0 | 0 | 0 |
| 1 | 10 | 13 | 19 | 15 |
| 2 | 19 | 23 | 30 | 25 |
| 3 | 24 | 31 | 38 | 32 |
| 4 | 28 | 37 | 43 | 37 |
| 5 | 31 | 42 | 47 | 42 |
| 6 | 33 | 45 | 51 | 45 |
| 7 | 35 | 48 | 54 | 48 |
| 8 | 36 | 50 | 57 | 49 |
| 9 | 37 | 51 | 59 | 51 |
| 10 | 38 | 53 | 60 | 52 |
| 11 | 39 | 54 | 61 | 53 |
| 12 | 40 | 55 | 62 | 54 |
| 13 | 40 | 56 | 63 | 55 |
| 14 | 41 | 56 | 63 | 56 |
| 15 | 41 | 57 | 64 | 56 |
| N | 4,782 | 11,271 | 6,027 | 22,080 |

Appendix Table 6. Cumulative percent of children ever again in a union, by maternal education and time elapsed since union disruption, 2003–2009, for children in Finland experiencing parental separation

| Duration | Maternal education | |  |  |
| --- | --- | --- | --- | --- |
| (years) | Low | Medium | High | All |
| 0 | 0 | 0 | 0 | 0 |
| 1 | 28 | 25 | 20 | 25 |
| 2 | 38 | 34 | 27 | 33 |
| 3 | 45 | 42 | 36 | 41 |
| 4 | 52 | 48 | 41 | 48 |
| 5 | 57 | 54 | 46 | 53 |
| 6 | 62 | 59 | 52 | 58 |
| 7 | 66 | 63 | 55 | 63 |
| 8 | 69 | 66 | 57 | 65 |
| 9 | 72 | 68 | 59 | 68 |
| 10 | 74 | 72 | 63 | 71 |
| N | 5,636 | 6,715 | 2,698 | 15,049 |

Appendix Table 7. Family type distributions at ages 0–15 years (in percent) by maternal education in Finland, 2003–2005

| Children of low educated mothers | | | | | | |
| --- | --- | --- | --- | --- | --- | --- |
|  |  |  |  |  |  |  |
| Age | With lone | With | With lone mother, | Mother and | With both | With both |
|  | mother | others | after union | stepfather | parents in | parents |
|  | from birth |  | dissolution |  | cohabitation | in marriage |
|  |  |  |  |  |  |  |
|  |  |  |  |  |  |  |
| 0 | 27 | 0 | 0 | 0 | 43 | 30 |
| 1 | 20 | 0 | 6 | 1 | 38 | 35 |
| 2 | 16 | 2 | 11 | 4 | 30 | 37 |
| 3 | 13 | 3 | 15 | 5 | 26 | 38 |
| 4 | 11 | 4 | 18 | 7 | 22 | 37 |
| 5 | 10 | 6 | 19 | 9 | 19 | 37 |
| 6 | 9 | 7 | 20 | 11 | 17 | 36 |
| 7 | 8 | 8 | 21 | 12 | 15 | 36 |
| 8 | 6 | 10 | 22 | 14 | 13 | 35 |
| 9 | 5 | 11 | 23 | 16 | 12 | 33 |
| 10 | 4 | 12 | 24 | 18 | 10 | 32 |
| 11 | 3 | 13 | 24 | 20 | 9 | 31 |
| 12 | 2 | 14 | 24 | 21 | 9 | 30 |
| 13 | 2 | 15 | 23 | 22 | 8 | 28 |
| 14 | 2 | 18 | 23 | 22 | 7 | 27 |
| 15 | 2 | 19 | 23 | 22 | 7 | 27 |
|  |  |  |  |  |  |  |
| N=11,251 | | | | | | |
|  |  |  |  |  |  |  |
| Children of medium educated mothers | | | | | | |
|  |  |  |  |  |  |  |
| Age | With lone | With | With lone mother, | Mother and | With both | With both |
|  | mother | others | after union | stepfather | parents in | parents |
|  | from birth |  | dissolution |  | cohabitation | in marriage |
|  |  |  |  |  |  |  |
|  |  |  |  |  |  |  |
| 0 | 12 | 0 | 0 | 0 | 39 | 48 |
| 1 | 9 | 0 | 2 | 0 | 34 | 54 |
| 2 | 7 | 0 | 5 | 2 | 29 | 57 |
| 3 | 6 | 1 | 8 | 3 | 25 | 58 |
| 4 | 5 | 1 | 9 | 4 | 21 | 60 |
| 5 | 5 | 2 | 11 | 5 | 18 | 59 |
| 6 | 5 | 2 | 12 | 7 | 15 | 59 |
| 7 | 4 | 2 | 13 | 9 | 13 | 58 |
| 8 | 4 | 3 | 14 | 10 | 12 | 56 |
| 9 | 3 | 4 | 15 | 11 | 11 | 56 |
| 10 | 3 | 4 | 16 | 13 | 10 | 54 |
| 11 | 2 | 5 | 16 | 14 | 9 | 54 |
| 12 | 2 | 6 | 17 | 16 | 8 | 52 |
| 13 | 2 | 7 | 17 | 16 | 7 | 50 |
| 14 | 1 | 9 | 17 | 18 | 6 | 49 |
| 15 | 1 | 10 | 17 | 19 | 6 | 47 |
|  |  |  |  |  |  |  |
| (N=28,398) | | | | | | |

(Continued)

| Children of highly educated mothers | | | | | | |
| --- | --- | --- | --- | --- | --- | --- |
|  |  |  |  |  |  |  |
| Age | With lone | With | With lone mother, | Mother and | With both | With both |
|  | mother | others | after union | stepfather | parents in | parents |
|  | from birth |  | dissolution |  | cohabitation | in marriage |
|  |  |  |  |  |  |  |
|  |  |  |  |  |  |  |
| 0 | 7 | 0 | 0 | 0 | 24 | 69 |
| 1 | 5 | 0 | 1 | 0 | 19 | 74 |
| 2 | 4 | 0 | 2 | 1 | 17 | 76 |
| 3 | 4 | 0 | 4 | 1 | 14 | 77 |
| 4 | 4 | 0 | 5 | 2 | 13 | 76 |
| 5 | 3 | 1 | 7 | 2 | 11 | 76 |
| 6 | 3 | 1 | 8 | 3 | 10 | 75 |
| 7 | 3 | 1 | 9 | 5 | 9 | 73 |
| 8 | 3 | 2 | 10 | 5 | 8 | 72 |
| 9 | 2 | 2 | 10 | 7 | 8 | 70 |
| 10 | 2 | 3 | 11 | 8 | 7 | 69 |
| 11 | 2 | 3 | 11 | 9 | 6 | 68 |
| 12 | 1 | 4 | 13 | 10 | 6 | 66 |
| 13 | 1 | 4 | 12 | 13 | 5 | 64 |
| 14 | 1 | 5 | 13 | 14 | 5 | 62 |
| 15 | 1 | 6 | 13 | 16 | 5 | 60 |
|  |  |  |  |  |  |  |
| N=24,513 | | | | | | |
|  |  |  |  |  |  |  |
| All children | | | | | | |
|  |  |  |  |  |  |  |
| Age | With lone | With | With lone mother, | Mother and | With both | With both |
|  | mother | others | after union | stepfather | parents in | parents |
|  | from birth |  | dissolution |  | cohabitation | in marriage |
|  |  |  |  |  |  |  |
|  |  |  |  |  |  |  |
| 0 | 12 | 0 | 0 | 0 | 32 | 55 |
| 1 | 9 | 0 | 2 | 0 | 28 | 60 |
| 2 | 7 | 0 | 5 | 1 | 24 | 62 |
| 3 | 6 | 1 | 7 | 3 | 20 | 63 |
| 4 | 6 | 1 | 9 | 4 | 18 | 63 |
| 5 | 5 | 2 | 11 | 5 | 15 | 62 |
| 6 | 5 | 3 | 12 | 6 | 14 | 61 |
| 7 | 5 | 3 | 13 | 8 | 12 | 59 |
| 8 | 4 | 4 | 14 | 9 | 11 | 57 |
| 9 | 3 | 5 | 15 | 11 | 10 | 55 |
| 10 | 3 | 6 | 17 | 13 | 9 | 53 |
| 11 | 2 | 7 | 17 | 15 | 8 | 51 |
| 12 | 2 | 8 | 18 | 16 | 8 | 48 |
| 13 | 2 | 10 | 19 | 18 | 7 | 45 |
| 14 | 2 | 12 | 19 | 19 | 6 | 43 |
| 15 | 2 | 13 | 19 | 20 | 6 | 40 |
|  |  |  |  |  |  |  |
| N=64,162 | | | | | | |
